# Combination antiretroviral therapy is associated with reduction in liver fibrosis scores in patients with HIV and HBV co-infection

**DOI:** 10.1186/s12981-021-00419-y

**Published:** 2021-12-19

**Authors:** Rongrong Yang, Xien Gui, Hengning Ke, Yong Xiong, Shicheng Gao

**Affiliations:** Department of Infectious Diseases, Zhongnan Hospital of Wuhan University, No.169, Donghu Road, Wuchang District, Wuhan, 430071 Hubei People’s Republic of China

**Keywords:** Liver fibrosis, Non-invasive assessment, FIB-4 index, Individuals with HIV/HBV co-infection, Antiretroviral therapy

## Abstract

**Background:**

Liver fibrosis is common in individuals with HIV/HBV co-infection, but whether cART could reverses liver fibrosis is unclear.

**Methods:**

This was a retrospective observational study. Binary logistic regression was used to assess predictors of liver fibrosis in individuals with HIV/HBV co-infection. Comparison of FIB-4 scores before and after cART were compared using *X*^*2*^ test and *t* test.

**Results:**

Four hundred and fifty-eight individuals with HIV/HBV co-infection were included in this study. It was found that cART (HR 0.016, 95% CI: 0.009–0.136; P < 0.001) was one of protection factors to against liver fibrosis. Forty individuals who had normal levels of ALT, AST and PLT during the whole course of diseases were stratified into FIB-4 < 1.45 (n = 14), 1.45 ≤ FIB-4 ≤ 3.25 (n = 19) and FIB-4 > 3.25 (n = 7) groups by their FIB-4 scores before cART. In 1.45 ≤ FIB-4 ≤ 3.25 group, 57.9%(11/19) of the individuals dropped to FIB-4 < 1.45 group by cART; in FIB-4 > 3.25 group, 85.7%(6/79) dropped to 1.45 ≤ FIB-4 ≤ 3.25 group, while 14.3%(1/7) dropped to FIB-4 < 1.45 group. In cART-naive group, 1 year, 2–5 years and 5–10 years post-cART groups, FIB-4 scores were 4.29 ± 0.43, 3.63 ± 0.38, 2.90 ± 0.36 and 2.52 ± 0.38, respectively (*P* = 0.034); and the incidence of liver fibrosis were 7.38%(104/141), 63.6%(98/154), 60.8%(62/102) and 47.5%(29/61), respectively (*P* = 0.004).

**Conclusion:**

cART was associated with decreased FIB-4 scores and the benefit of cART in reversing liver fibrosis can sustain for a decade in patients with HIV/HBV co-infection.

**Supplementary Information:**

The online version contains supplementary material available at 10.1186/s12981-021-00419-y.

## Introduction

In human immunodeficiency virus (HIV)-infected individuals, chronic hepatitis B virus (HBV) infection is common due to shared modes of transmission, and accounts for the majority of liver disease in individuals with HIV [1] infection. Published studies have also shown that HIV can accelerate the progression of viral hepatitis and related liver disease, including liver fibrosis, cirrhosis, and hepatocellular carcinoma [2–4]. Liver fibrosis is an accumulation of extracellular matrix in the liver in response to various chronic liver injuries. A study suggested that HIV infection was associated with increased aspartate aminotransferase (AST)-to-platelet (PLT) ratio index (APRI), a surrogate marker for hepatic fibrosis [5]. A recent study by Kong et al. suggested that entecavir can lead to histological reversal of fibrosis in chronic hepatitis B patients [6]. However, data about fibrosis prevalence and combination antiretroviral therapy (cART) response in individuals with HIV/HBV co-infection in China are limited.

In this study, the prevalence and risk factors for hepatic fibrosis in individuals with HIV/HBV co-infection, as measured by Fibrosis-4 (FIB-4) index, were evaluated. Whether cART could reverse the fibrogenic effect of HIV infection was also demonstrated.

## Methods

### Study design and population

Individuals with HIV/HBV co-infection were screened for eligibility. The inclusion criteria were laboratory assessment allowing FIB-4 calculation (AST, ALT, platelet count) performed on the same day; hepatitis B surface antigen (HBsAg) positivity for at least 6 months before screening; confirmed with HIV infection. The exclusion criteria were decompensated cirrhosis; HAV, HCV and HEV infection; heavy alcohol consumption (40 g pure alcohol per day); other liver comorbidity including hemochromatosis, Wilson’s disease, 1-antitrypsin deficiency, autoimmune hepatitis, alcoholic or nonalcoholic steatohepatitis; any complications of severe heart, lung, kidney, brain, or blood diseases or other important systematic diseases; severe neurological or psychological disease; and pregnant or lactating women,as reported previously [7].

This study was conducted in accordance with the principles of the Declaration of Helsinki. The protocol was approved by the Institutional Review Board of Zhongnan Hospital of Wuhan University. Informed consent was obtained from all participants.

### Virological and biochemical assays

Serum HBsAg, anti-HBs, HBeAg, anti-HBe and anti-HBc were measured by electrochemiluminescence immunoassay (Architect i2000SR, ABBOTT, Wiesbaden, Germany). Serum HBV DNA levels were determined in the central laboratory by COBAS® TaqMan® HBV Test (Roche Molecular Systems, Branchburg, NJ, USA) with a detectable level of 20 IU/mL. Haematology and serum biochemical markers, including platelet count (PLT), alanine aminotransferase (ALT) and aspartate aminotransferase (AST) were measured by autoanalyser according to the manufacturer’s instructions.

### Noninvasive measurement of liver fibrosis

Serum AST and ALT activities were routinely measured; usual upper normal values were 35 IU/l and 45 IU/l, respectively. Platelet counts were performed in the same day; normal values were (100–300) × 10^9^/l. The FIB-4 values were calculated automatically using the formula: age (years) × AST [U/l]/(platelets [10^9^/l] × (ALT [U/l])^1/2^). As a previous study [8], FIB-4 < 1.45 was considered as no or moderate fibrosis (F0-F1-F2-F3), and FIB-4 > 3.25 was considered as extensive fibrosis or cirrhosis (F4-F5-F6) (in the ISHAK classification of fibrosis). FIB-4 was used when analyzing the incidence of liver fibrosis and FIB-4 > 3.25 was considered as liver fibrosis in this study.

### Statistical analysis

Descriptive statistics including mean ± SD, counts and percentages were used to describe the demographics and clinical characteristics of the patients. Independent sample *t* tests or MannWhitney *U* test was conducted to compare continuous variables. Paired *t* test was used to compare FIB-4 scores and associated indexes of the same patients before and after cART. One-Way ANOVA was used to compare FIB-4 scores of patients at different duration of cART. Chi-square or Fisher’s exact test was used to compare categorical variables. All calculations were made using SPSS (version 11.0.4.0) software (SPPS Inc., Chicago, IL). A *P* value of less than 0.05 was considered statistically significant.

## Results

### Baseline characteristics

A total of 458 individuals with HIV/HBV co-infection were included in this study (Table 1). The mean age was 46 years, and 362 (79.0%) were male. Their mean BMI was 21.37, and the proportion of alcohol use was 2.8%(13/458). The mean levels of ALT, AST and platelets were 42.8 IU/L, 44.7 IU/L and 159 × 10^9^/L. The mean FIB-4 scores was 4.869 and 65.9% of the study population had FIB-4 scores ≥ 1.45. 57.2% of the study population had CD4^+^ T lymphocyte count < 200 cells/ul. 31.7% (145/458) individuals had detectable HIV RNA (> 20 copies/mL) and 97.2%(141/145) of them didn’t received cART. 41.3% (189/458) had detectable HBV DNA (> 20 copies/mL), and 66.1%(125/189) of them were overlap with HIV-RNA positive. 27.3% was positive for HBeAg, and 41.3% was positive for anti-HBe.

**Table 1:** Baseline characteristics of the study population

Characteristic	HIV/HBV co-infected (n = 458)
Male (n, %)	362(79.0)
Age Years, Mean ± SD	46 ± 12
BMI, Mean ± SD	21.37 ± 2.43
Alcohol use, yes (n, %)	13(2.8)
ALT IU/L, Mean ± SD	42.8 ± 4.3
AST IU/L, Mean ± SD	44.7 ± 4.9
Platelets 10^9^/L, Mean ± SD	159 ± 4
FIB-4, Mean ± SD	4.869 ± 0.913
< 1.45(n, %)	156(34.1)
1.45–3.25(n, %)	164(35.8)
> 3.25(n, %)	138(30.1)
CD4 cell count, Mean ± SD	161 ± 11
CD4 < 200 cells/ul(n, %)	262(57.2)
CD4 ≥ 200 cells/ul(n, %)	196(42.8)
HIV RNA > 20 copies/ml(n, %) *	145(31.7)
HBV DNA > 20 copies/ml(n, %) §	189(41.3)
HBeAg positive(n, %)	125(27.3)
Anti-HBe positive(n, %)	189(41.3)
Treatment history	
Months of HIV diagnosis, Mean ± SD	38.03 ± 2.50
Months of ART treatment, Mean ± SD	35.89 ± 4.72
TDF/3TC based regimen (n, %)	246/317 (77.6)
AZT/3TC based regimen (n, %)	71/317 (22.4)

^*^The 145 individuals who have detectable VL, 141 (30.8%) individuals didn’t received ART treatment. The remaining 4 individuals received ART, but the HIV-RNA levels were low (46 copies/ml; 52 copies/ml; 106 copies/ml and 896 copies/ml, respectively). §Among the 189 individuals, 66.1%(125/189) of them were overlap with HIV-RNA positive. In HBV DNA + /HIV-RNA + and HBV DNA + /HIV-RNA- group, FIB-4 were 5.152 ± 0.551 and 4.403 ± 0.576, respectively. The difference between the two groups was not statistically significant (t = 0.817, P = 0.414)

### Risk factors for liver fibrosis in patients with HIV/HBV co-infection according to FIB-4

A total of 13 parameters were included in univariate analysis for the risk of liver fibrosis in individuals with HIV/HBV co-infection, including demographic indicators (such as gender and age), HIV associated indicators (such as whether cART, whether HIV-RNA detectable, CD4 levels), HBV associated indicators (such as HBsAg titer, HBeAg and HBeAb status, whether HBV-DNA detectable), ALT, AST and PLT levels. Those variables with *P* < 0.1 were included in subsequent multivariate analysis (Table 2).

**Table 2:** Risk factors associated with liver fibrosis (FIB-4) in patients with HIV/HBV co-infection

	Univariate		Multivariate	
	*P*	OR(95% CI)	*P*	OR(95% CI)
Male	0.706	0.912(0.565–1.473)		
≥ 45 years	0.000	4.103(2.726–6.174)	0.000	7.194(3.259–15.884)
HCV co-infection	0.038	2.149(1.042–4.430)	0.147	3.155(0.668–14.899)
HBsAg < 50	0.201	0.673(0.366–1.236)		
HBeAg positive	0.002	2.075(1.314–3.279)	0.009	0.158(0.040–0.634)
HBeAb positive	0.003	2.044(1.280–3.262)	0.569	1.402(0.438–4.484)
Detectable HBV-DNA	0.001	2.201(1.404–3.451)	0.309	1.759(0.592–5.225)
Detectable HIV-RNA	0.001	2.081(1.337–3.239)	0.000	27.850(1.607–81.401)
ART	0.005	0.532(0.341–0.829)	0.000	0.016(0.009–0.136)
CD4 > 200/ul	0.000	0.300(0.188–0.479)	0.012	0.336(0.144–0.786)
ALT > 50 U/l	0.005	3.929(1.508–10.242)	0.046	20.488(1.012–60.190)
AST > 40 U/l	0.000	20.941(5.054–86.773)	0.026	27.323(1.478–55.122)
PLT < 100(× 10^9^/l)	0.000	13.901(4.351–75.232)	0.000	61.246(7.361–109.604)

At multivariate analysis, older than 45 years (HR 7.194, 95% CI: 3.259–15.884; P < 0.001), ALT > 50 U/l (HR 20.488, 95% CI: 1.012–60.190; P = 0.046), AST > 40 U/l (HR 27.323, 95% CI: 1.478–55.122; P = 0.026), PLT < 100(× 10^9^/l) (HR 61.246, 95% CI: 7.361–109.604; P < 0.001) and detectable HIV-RNA (HR 27.850, 95% CI: 1.607–81.401; P < 0.001) were risk factors for liver fibrosis in individuals with HIV/HBV co-infection. Also, HBeAg positive (HR 0.158, 95% CI: 0.040–0.634; P = 0.009), cART (HR 0.016, 95% CI: 0.009–0.136; P < 0.001) and CD4 > 200/ul (HR 0.336, 95% CI: 0.144–0.786; P = 0.012) were protective factors to against liver fibrosis in individuals with HIV/HBV co-infection.

### Effect of risk factors on the incidence of liver fibrosis in patients with HIV/HBV co-infection

The incidence of liver fibrosis and relative risk by different factors in individuals with HIV/HBV co-infection were shown (Table 3). The incidence of liver fibrosis in patients ≥ 45 years were 3.688-fold higher than patients < 45 years (76.5 vs 46.9%, *P* < 0.001). Compared with patients positive for HBeAg, those patients negative for HBeAg were 2.004-fold more likely to have liver fibrosis (*P* = 0.002). Also, the incidence of liver fibrosis in patients who had detectable HIV-RNA were 1.975-fold higher than patients who had undetectable HIV-RNA (73.3 vs 58.2%, *P* = 0.001). The relative risk between cART and cART-naive patients was 1.914, between patients had CD4 ≤ 200/ul and CD4 > 200/ul was 3.803. Patients had ALT > 50U/l, AST > 40U/l and PLT < 100(× 10^9^/l) were 5.079-fold, 8.428-fold and 8.415-fold more likely to have liver fibrosis, respectively, compared to those had normal levels of ALT, AST and PLT.

**Table 3:** Incidence of liver fibrosis by different risk factors in patients with HIV/HBV co-infection

Group	Number	No. of liver fibrosis (%)	*X*^*2*^	*P*	Relative risk (95% *CI*)
≥ 45 Years	264	202(76.5)	42.534	< 0.001	3.688(2.470–5.505)
< 45 Years	194	91(46.9)			
HBeAg(-)	358	242(67.6)	9.343	0.002	2.004(1.278–3.144)
HBeAg( +)	100	51(51.0)			
Detectable HIV-RNA	176	129(73.3)	10.777	0.001	1.975(1.312–2.973)
Unetectable HIV-RNA	282	164(58.2)			
ART-naive	148	109(73.6)	8.880	0.003	1.914(1.245–2.943)
ART	310	184(59.4)			
CD4 ≤ 200/ul	322	236(73.3)	40.853	< 0.001	3.803(2.497–5.792)
CD4 > 200/ul	136	57(41.9)			
ALT > 50U/l	89	78(87.6)	26.846	< 0.001	5.079(2.614–9.869)
Normal ALT	369	215(58.3)			
AST > 40U/l	149	134(89.9)	64.573	< 0.001	8.428(4.726–15.028)
Normal AST	309	159(51.5)			
PLT < 100(× 10^9^/l)	121	110(90.9)	51.766	< 0.001	8.415(4.368–16.214)
Normal PLT	337	183(54.3)			

### Effect of cART on FIB-4 scores and associated indexes in 212 patients with HIV/HBV co-infection

In this study, 212 individuals with HIV/HBV co-infection had FIB-4 scores and associated indexes before and after cART (Table 4). FIB-4 declined from 2.54 ± 0.77 to 1.57 ± 0.23 (*P* < 0.001) by cART, and the proportion of liver fibrosis declined from 81.1 to 56.6% (*P* < 0.001). Also, the levels of AST and PLT improved after cART. The proportion of patients who had elevated ALT and AST, and patients who had thrombocytopenia were reduced after cART.

**Table 4:** Comparison of FIB-4 scores and associated indexes before and after ART in 212 patients with HIV/HBV co-infection

	ART-naive	ART	Test	*P*
FIB-4	2.54 ± 0.77	1.57 ± 0.23	3.899	< 0.001
Proportion of liver fibrosis(n,%)	172(81.1)	120(56.6)	29.745	< 0.001
ALT U/l (mean ± SD)	49 ± 6	36 ± 7	1.490	0.142
ALT > 50U/l (n,%)	64(30.2)	28(13.2)	17.991	< 0.001
AST U/l (mean ± SD)	68 ± 8	37 ± 4	4.095	< 0.001
AST > 40U/l (n,%)	120(56.6)	52(24.5)	45.233	< 0.001
PLT < 100(× 10^9^/l) (mean ± SD)	133 ± 9	191 ± 12	-4.816	< 0.001
PLT < 100(× 10^9^/l) (n,%)	64(30.2)	20(9.4)	28.742	< 0.001

### Effect of cART on FIB-4 scores and grades in HIV/HBV co-infected patients with normal levels of ALT, AST and PLT

Liver enzyme elevation and thrombocytopenia are common among individuals with HIV infection. As ALT, AST and platelet values are used in FIB-4 calculation, higher FIB-4 scores in patients with HIV infection may be due to HIV-related liver injuries or thrombocytopenia rather than hepatic fibrosis. To further explore this, those 40 individuals with HIV/HBV co-infection who had normal levels of ALT, AST and PLT during the whole course of diseases were selected.

**Fig. 1:**
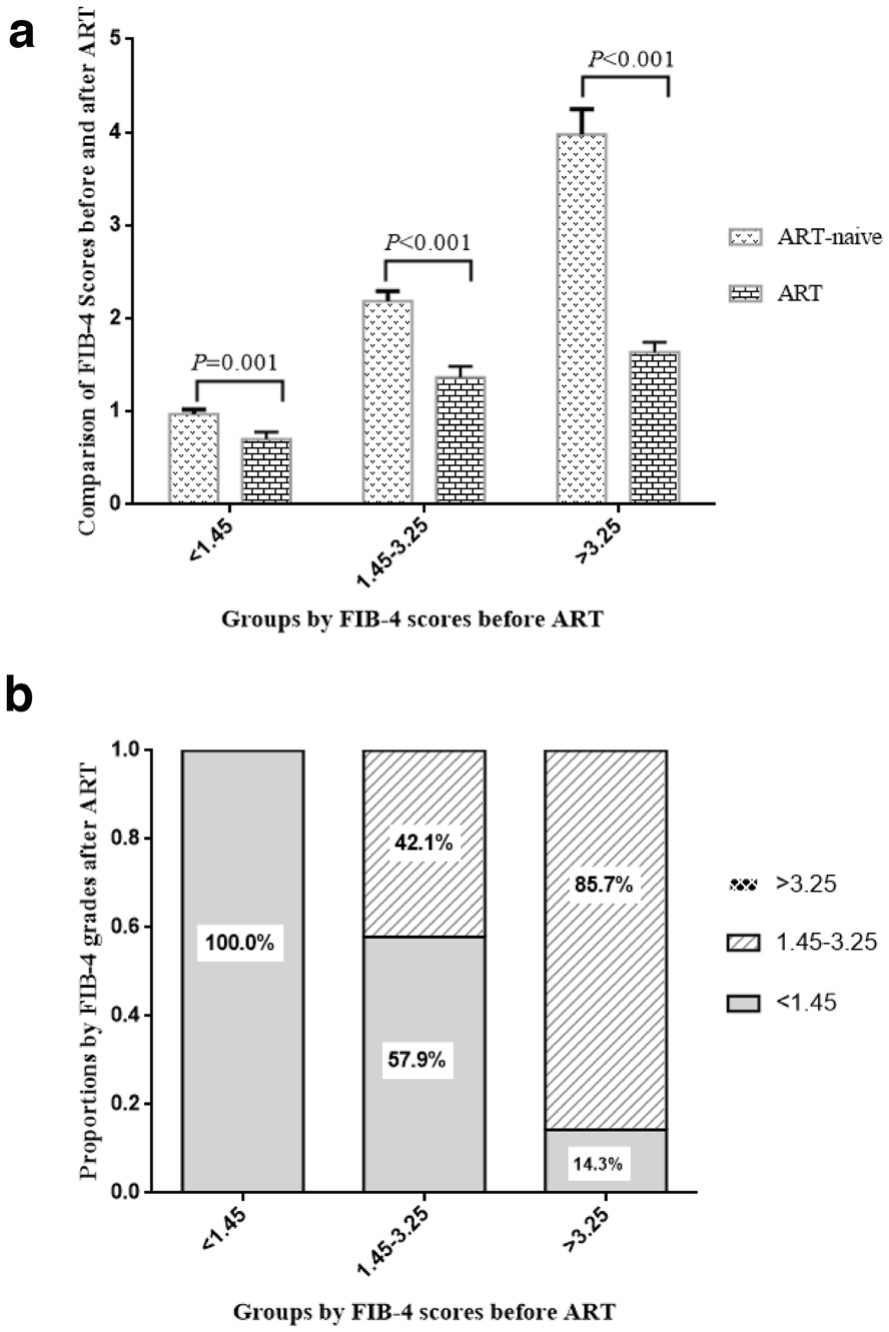
Effect of ART on FIB-4 scores and grades in HIV/HBV co-infected patients with normal levels of ALT, AST and PLT

The 40 individuals with HIV/HBV co-infection were stratified into FIB-4 < 1.45 (n = 14), 1.45 ≤ FIB-4 ≤ 3.25 (n = 19) and FIB-4 > 3.25 (n = 7) groups by their FIB-4 scores before cART. In FIB-4 < 1.45 group, the scores declined from 0.970 ± 0.052 before cART to 0.701 ± 0.080 by cART (*P* = 0.001, Fig. 1a); all the 14 individuals with HIV/HBV co-infection maintained FIB-4 < 1.45 grade after cART (Fig. 1b). Interestingly, in 1.45 ≤ FIB-4 ≤ 3.25 group, the scores declined from 2.187 ± 0.109 to 1.369 ± 0.115 by cART (*P* < 0.001, Fig. 1a); FIB-4 grade in 57.9%(11/19) of the individuals with HIV/HBV co-infection dropped to a lower FIB-4 grade (FIB-4 < 1.45) by cART (Fig. 1b). Moreover, in FIB-4 > 3.25 group, the scores declined from 3.978 ± 0.270 to 1.636 ± 0.111 after cART (*P* < 0.001, Fig. 1a); FIB-4 grade in 85.7%(6/79) of the individuals with HIV/HBV co-infection dropped to 1.45 ≤ FIB-4 ≤ 3.25 grade, while 14.3%(1/7) dropped to FIB-4 < 1.45 grade after cART (Fig. 1b).

### Effect of cART duration on FIB-4 scores and incidence of liver fibrosis

**Fig. 2:**
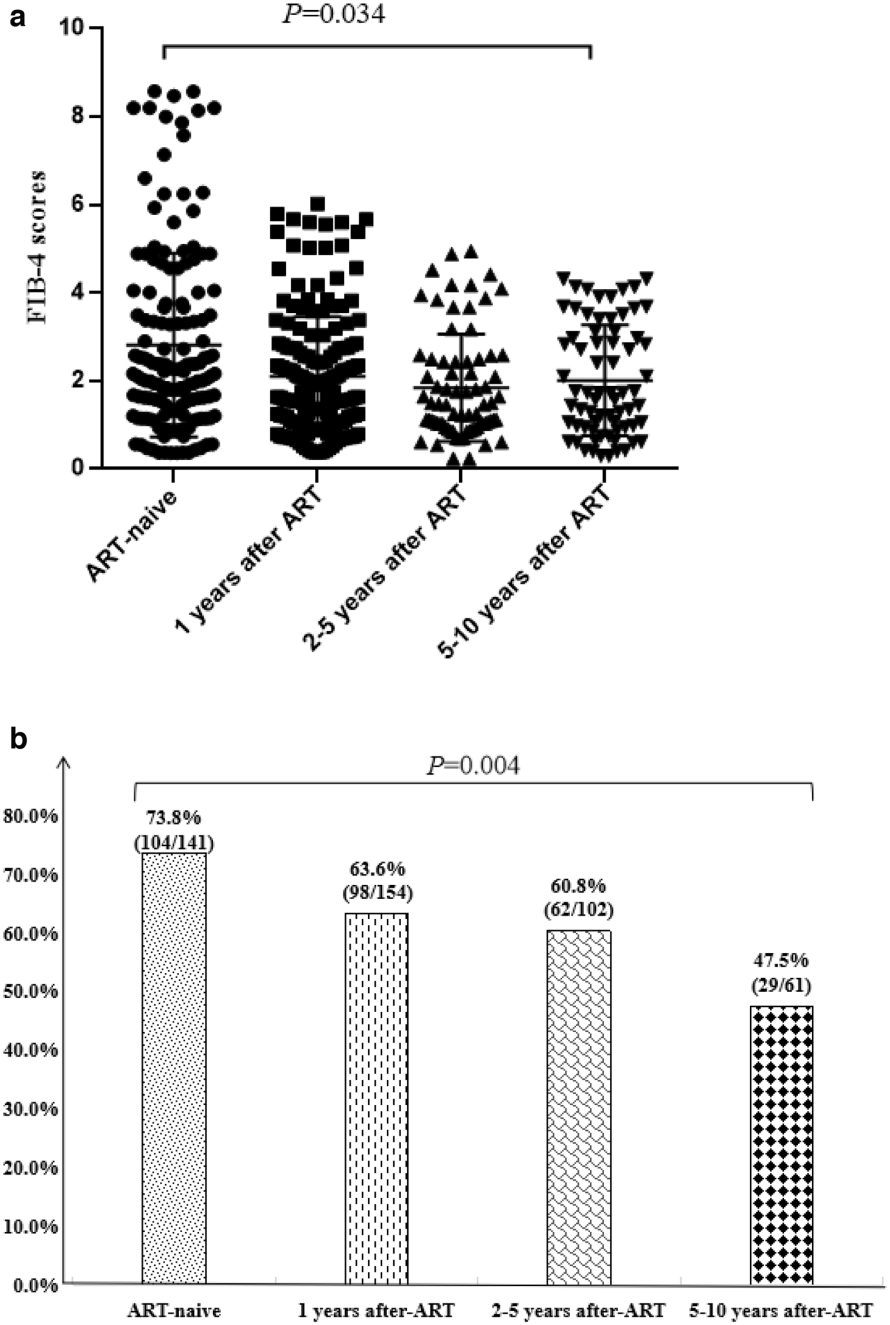
Effect of ART duration on FIB-4 scores and the incidence of liver fibrosis

According to cART duration, all the patients with HIV/HBV co-infection in this study were divided into cART-naive group, 1 year, 2–5 years and 5–10 years after cART groups. In these four groups, the FIB-4 scores were 4.29 ± 0.43, 3.63 ± 0.38, 2.90 ± 0.36 and 2.52 ± 0.38, respectively, which showed statistically significant differences (*P* = 0.034) (Fig. 2a).

Moreover, the incidence of liver fibrosis in cART-naive group, 1 year, 2–5 years and 5–10 years after cART groups were 73.8%(104/141), 63.6%(98/154), 60.8%(62/102) and 47.5%(29/61), respectively. The difference of incidence of liver fibrosis between the four groups was statistically significant (*P* = 0.004) (Fig. 2b).

## Discussion

The incidence and predictors of liver fibrosis progression estimated by FIB-4 index in individuals with HIV/HBV co-infection were evaluated in this study. Most importantly, we found that higher levels of HIV-RNA and lower CD4 cell count tended to be associated with an increased risk for higher FIB-4 scores by the multivariate analysis. Furthermore, it was interestingly that the initiation of cART was associated with a significant reduction in fibrosis scores, which provide further support for early initiation of cART in individuals with HIV/HBV co-infection.

In this study, the results of multivariate analysis showed that variables related with HIV natural history (such as levels of HIV RNA and CD4^+^ T-cell count) and HIV-associated intervention (such as cART) could affect FIB-4 scores and the incidence of liver fibrosis. The fact, that HIV infection plays an important role in the development of hepatic fibrosis [5], was reconfirmed in this study. The biological explanation for this finding is unknown, but there are several possibilities. In addition to direct infection of hepatic stellate cells, which can express C–C chemokine receptor type 5 (CCR5) and are considered as a source of fibrogenesis [2, 9], HIV-related immune activation [10] and increased intrahepatic apoptosis [11] may also hasten the progression of liver fibrosis. Didanosine, which was still be prescribed in resource-limited settings, was confirmed to have a lasting negative effect on liver fibrosis in those with HIV for its insulin resistance and mitochondrial toxicity [12, 13]. In March 2005, didanosine was no longer as a regimen of cART for AIDS in China. In this study, the negative effect of didanosine on liver fibrosis was excluded. A higher HIV-1 RNA viral load and a lower CD4^+^ T-lymphocyte count have both been associated with a higher FIB-4 in HIV-infected individuals without chronic HBV or HCV infection [14–17]. Some studies have reported associations between microbial translocation-related immune activation and accelerated liver fibrosis in HIV-HCV coinfection [18–21] and HIV monoinfection [22], which suggested that microbial translocation may be an important mechanism through which HIV accelerates liver fibrosis. In this study, cART was found to show protective role against liver fibrosis. We speculated that the relatively confirmed reason may be related to the reduction of HIV-1 RNA viral load and the increase of CD4^+^ T-lymphocyte count level by cART, and the inhibition of microbial translocation and immune activation after cART may also be another reason for the improvement with FIB-4.

The result of CD4 > 200/ul as a protective factor to against liver fibrosis in this study supports a mechanism of immune activation, but detectable HIV-RNA as a risk factor for liver fibrosis supports a mechanism of a direct effect of HIV infection. Taken together, we believe that HIV infection accelerates the occurrence of liver fibrosis through the synergistic effect of direct infection by HIV and HIV-related immune activation.

A published study aimed HIV/HCV co-infection patients have shown that although patients seem to be protected by cART especially in the first years after its prescription, liver fibrosis progressed faster afterwards, making HIV/HCV-coinfected patients a population that should be prioritized for access to HCV treatment [23]. However, the situation of individuals with HIV/HBV co-infection is different from that of HIV/HCV co-infected persons. cART with dual activity against HIV and HBV was recommended as an ideal choice for patients with individuals with HIV/HBV co-infection. In this study, we found that with lifelong cART containing anti‐HBV agents, both the marker for hepatic fibrosis (estimated by FIB-4 in this study) and the incidence of liver fibrosis decreased with the prolongation of time on treatment. The result was consistent with a French study, which concluded that long-term tenofovir use was associated with a decrease in fibrosis scores [24]. A previous study used liver stiffness measurement (LSM) by transient elastography (TE) to analyze the effect of HIV infection and TDF-containing cART on hepatic fibrosis both in patients with HIV alone and HIV-HBV coinfection. It was found that initiation of TDF-containing cART was associated with reduced liver fibrosis/cirrhosis, and many HIV-HBV coinfected patients had significant fibrosis/cirrhosis at 1 year on cART [25]. In our study, FIB-4 was used to estimate liver fibrosis in 458 individuals with HIV-HBV coinfection, and 63.6% individuals were found to have liver fibrosis at 1 year after cART. The two studies used different evaluation indexes and found similar results that cART could reduce the incidence of liver fibrosis in individuals with HIV-HBV coinfection. The difference of our study was that comparative observation was also conducted on individuals who had been treated with cART for 2–5 years and 5–10 years, which is a supplement to the published paper. Therefore, we conclude that timely cART, in addition to reducing AIDS-related morbidity and mortality, is also an effective intervention to reduce the occurrence of liver related complications in people living with HIV and HBV co-infection.

Except for HIV-associated indicators, impaired liver function and thrombocytopenia caused by hypersplenism are characteristics for patients with advanced liver disease, and levels of AST, ALT and platelet count can directly affect FIB-4 scores, which was used as a noninvasive tests to assess hepatic fibrosis. cART itself also known to cause improvement in AST/ALT/platelets in HIV individuals and that may not necessarily reflect it is improving liver fibrosis progress. To reduce the confounding effect of the above factors, those individuals who had normal levels of ALT, AST and PLT were selected and analyzed FIB-4 scores changes before and after cART. In HIV individuals who had normal levels of ALT, AST and PLT before cART, we confirmed that the differences in FIB-4 after cART may not due to the recovery of liver function by the drugs with anti-HBV activity or the reconstruction of platelets after cART (Additional file 1: Figure S1).

Also, older age and negative status of HBeAg were found to be risk factors for liver fibrosis. This was in keeping with other studies that have used non-invasive measures to determine the extent of fibrosis in individuals with HIV/HBV co-infection [5, 26, 27]. Moreover, positive status of HBeAg was found to be a protective factor to against liver fibrosis in this study. The background of HBV infection in China is different from European and American countries, where individuals acquired HBV infection through sexual activity or injection drug use during adulthood. In this study, chronic HBV infection mostly occurred during childhood or perinatal period. Both older age and negative status of HBeAg indirectly indicates that the individuals has been infected with HBV for relatively longer time, which is associated with a higher risk of liver fibrosis. This is the natural history of HBV infection.

We recognize that our study has limitations. An inherent limitation of this study, first of all, was that serum markers (FIB-4) as a surrogate for the amount of liver fibrosis, which couldn't possibly represent the results by liver biopsy [28]. Although meta-analyses demonstrate that FIB-4 have moderate accuracy for assessing liver fibrosis [29], the limitation of using FIB-4 to predict liver fibrosis should be acknowledged to some extent. Second, as the cross-sectional nature of the study, we were not able to obtain data before and after cART for all individuals. However, similar results were found by comparing FIB-4 scores of 52 patients whose serum markers were available before and after cART. Finally, since HIV infection is associated with reduced PLT [30] and FIB-4 scores may be elevated due to HIV infection; therefore, we did a sensitivity analysis using only AST as a surrogate fibrosis marker, and found similar results.

In summary, this study suggests that HIV-associated indicators can predict the risk of liver fibrosis in individuals with HIV/HBV co-infection. As a noninvasive tests to assess hepatic fibrosis, FIB-4 scores decreased after cART until 5–10 years after cART.

## Supplementary Information

**Additional file 1**: **Figure S1.**. The correlation trend between FIB-4 and LSM.

## Data Availability

The datasets used or analysed during the current study are available from the corresponding author on reasonable request.
