# Supplementary figures and images for: Combination antiretroviral therapy is associated with reduction in liver fibrosis scores in patients with HIV and HBV co-infection
Source: AIDS Res Ther. 2021 Dec 19;18:98. doi: 10.1186/s12981-021-00419-y (PMC8684625; doi:10.1186/s12981-021-00419-y)

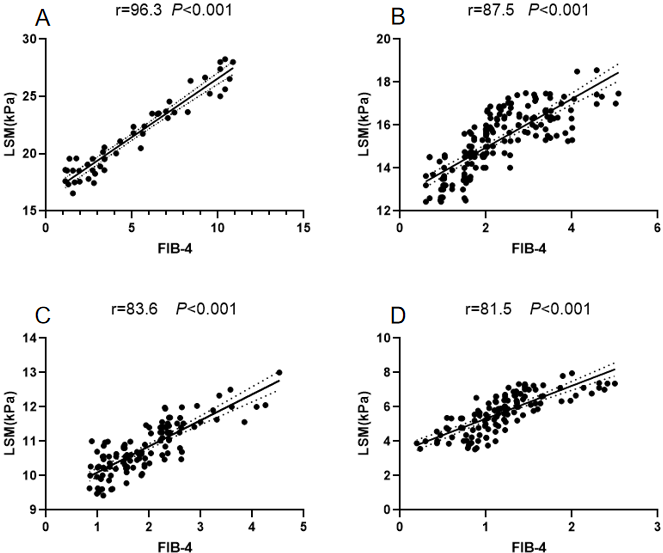


Figure S1. The correlation trend between FIB-4 and LSM

Supplement: Supplementary file 1 — Additional file 1: Figure S1.. The correlation trend between FIB-4 and LSM. [file 12981_2021_419_MOESM1_ESM.doc]
